# Validating the Cognitive Scale for Down Syndrome (CS-DS) to Detect Longitudinal Cognitive Decline in Adults With Down Syndrome

**DOI:** 10.3389/fpsyt.2019.00158

**Published:** 2019-04-16

**Authors:** Carla M. Startin, Bryony Lowe, Sarah Hamburg, Rosalyn Hithersay, Andre Strydom, LonDownS Consortium, Andre Strydom

**Affiliations:** ^1^Department of Forensic and Neurodevelopmental Sciences, Institute of Psychiatry, Psychology and Neuroscience, King's College London, London, United Kingdom; ^2^Division of Psychiatry, University College London, London, United Kingdom; ^3^LonDownS Consortium, London, United Kingdom

**Keywords:** Down Syndrome, dementia, cognitive decline, informant rating, cognitive scale for Down Syndrome (CS-DS)

## Abstract

Down syndrome (DS) is associated with intellectual disability and an ultra-high risk of developing dementia. Informant ratings are invaluable to assess abilities and related changes in adults with DS, particularly for those with more severe intellectual disabilities and/or cognitive decline. We previously developed the informant rated Cognitive Scale for Down Syndrome (CS-DS) to measure everyday cognitive abilities across memory, executive function, and language domains in adults with DS, finding CS-DS scores are a valid measure of general abilities, and are significantly lower for those with noticeable cognitive decline compared to those without decline. To further test the validity of the CS-DS in detecting changes associated with cognitive decline we collected longitudinal data across two time points, approximately 1.5–2 years apart, for 48 adults with DS aged 36 years and over. CS-DS total scores (78.83 ± 23.85 vs. 73.83 ± 25.35, *p* = 0.042) and executive function scores (46.40 ± 13.59 vs. 43.54 ± 13.60, *p* = 0.048) significantly decreased between the two time points, with scores in the memory domain trending towards a significant decrease (22.19 ± 8.03 vs. 20.81 ± 8.63, *p* = 0.064). Adults with noticeable cognitive decline at follow-up showed a trend to significantly greater change in total scores (7.81 ± 16.41 vs. 3.59 ± 16.79, *p* = 0.067) and significantly greater change in executive function scores (5.13 ± 9.22 vs. 1.72 ± 9.97, *p* = 0.028) compared to those without decline. Change in total scores showed significant correlations with change in scores from other informant measures of everyday adaptive abilities and symptoms associated with dementia, and participant assessment of general cognitive abilities (all *p* < 0.005), while change in memory scores (*R*^2^ = 0.28, *p* = 0.001) better predicted change in participant cognitive assessment scores than change in executive function (*R*^2^ = 0.15, *p* = 0.016) or language (*R*^2^ = 0.15, *p* = 0.018) scores. These results suggest informants may better detect changes in the executive function domain, while change in informant rated memory scores best predicts change in assessed cognitive ability. Alternatively, memory domain scores may be sensitive to changes across both early and late cognitive decline, whereas executive function domain scores are more sensitive to changes associated with later noticeable cognitive decline. Our results provide further support for the validity of the CS-DS to assess everyday cognitive abilities and to detect associated longitudinal changes in individuals with DS.

## Introduction

Down Syndrome (DS), caused by the triplication of chromosome 21, has a UK prevalence of approximately 1 in 1,000 live births (1). DS is the most common genetic cause of intellectual disability (ID), and is also associated with an ultra-high risk of developing dementia due to Alzheimer's disease (2); lifetime dementia risk may be as high as 95% (3). There is large variability in these phenotypes however, with ID severity varying from mild to severe/profound, and the age of dementia onset varying from the late 30 s to the 60 s (4, 5).

This variability in cognitive abilities and high rate of dementia in individuals with DS poses a challenge to the development of cognitive test batteries that are suitable for all individuals. While several batteries have been developed for use in adults with DS (5–9), some individuals are unable to complete tasks or perform at floor level, in particular older adults who are showing cognitive decline. For these individuals, informant ratings are invaluable to assess abilities and related changes. Although previously published questionnaires exist to assess symptoms related to dementia (10–12), a lack of a dedicated informant measure assessing everyday cognitive abilities suitable for the majority of people with DS led us to develop the Cognitive Scale for Down Syndrome (CS-DS) (13). The CS-DS focuses on abilities related to memory, executive function, and language, as these are often impaired in people with DS (14–18). We found the CS-DS is suitable for use with a wide range of individuals with DS, showing a range of scores and minimal floor and ceiling effects. It shows high inter-rater and test-retest reliability, with scores correlating well with measures of general abilities in adults without cognitive decline, and lower scores for those with significant cognitive decline compared to those without decline.

To further test the validity of the CS-DS in detecting changes associated with cognitive decline in those with DS, we collected longitudinal data with the CS-DS completed at two time points, ~1.5–2 years apart, for adults with DS aged 36 years and over. Here we present longitudinal analysis exploring changes in total and domain scores over this time.

## Materials and Methods

### Participants

Of the 128 adults with DS in the original CS-DS study (13), all 63 adults aged 36 years and over were contacted for a follow-up assessment ~1.5–2 years after their original assessment. Adults aged 36 years and over were contacted for a follow-up assessment due to the increased risk of cognitive decline in this age group, based on previous studies investigating cognitive decline in adults with DS and neuropathological studies indicating the presence of Alzheimer's disease pathology in the brains of almost all adults with DS from the mid-30s (2); adults aged 35 and under were not expected to show cognitive decline and so did not have a follow-up assessment. All participants had originally been recruited from across England from the LonDownS adult cohorts; see Startin et al. (5) for full details of the cohorts and assessment battery.

### Assessment

Informants for participants with DS were asked to complete the CS-DS (13) at both the original and follow-up assessments. All informants knew the participant well, and were usually relatives or paid carers. The CS-DS consists of 61 questions with three options for informants to select from: never/rarely true, sometimes true, or often/always true. Half the questions are reverse phrased to reduce response bias, with scores of 0, 1, or 2 depending on the response (higher scores indicate higher abilities). CS-DS total scores and scores for the memory, executive function, and language domains were used in analysis.

In addition to informants completing the CS-DS, they completed the short Adaptive Behavior Scale (short ABS) and the Dementia Questionnaire for People with Learning Disabilities (DLD). The short ABS assesses everyday adaptive abilities (with higher scores indicating higher abilities) (19), while the DLD assesses cognitive and social symptoms associated with dementia (with higher scores indicating poorer abilities) (11). Participants who met vision and hearing thresholds [see Startin et al. (5)] were administered the Kaufman Brief Intelligence Test 2 (KBIT-2), a measure of general cognitive abilities (20); seven individuals were not administered the KBIT-2 due to not meeting the vision or hearing threshold. The KBIT-2 contains two subscales, a verbal scale (verbal knowledge and riddle completion tests) and a non-verbal scale (pattern completion test). Each test was started at question 1 and stopped after four consecutive incorrect answers. Total raw scores were used for analysis due to high floor effects when converting to age adjusted IQ scores (5).

Informants were also administered the Cambridge Examination of Mental Disorders of Older People with Down's Syndrome and Others with Intellectual Disabilities (CAMDEX-DS) (21) to assess the presence or absence of noticeable cognitive decline. Noticeable cognitive decline was defined as decline occurring in the memory domain and in either the other cognitive functions or personality and behaviour domains (therefore indicating decline in multiple domains), with the decline not co-occurring with other factors such as depression.

### Statistical Analysis

We used SPSS version 22 for all analyses. Missing items on the CS-DS were given a score of one (the median possible score) when up to five answers were missing, while missing items on the DLD were imputed for up to 15% of items within each domain using the nearest integer to the mean value of completed scores within that domain.

To determine whether CS-DS scores changed over time, we first compared CS-DS scores at the two time points using paired samples *t*-tests; total scores were compared, in addition to scores for the memory, executive function, and language domains. Given the presence of Alzheimer's disease neuropathology in adults with DS by the mid-30s (2, 22), cognitive decline may have occurred in all assessed individuals, whether or not this had been noticed by carers, and so analysis was performed across the whole group to determine whether a significant change in scores had occurred. In addition, as changes in CS-DS scores may be greater in those with noticeable cognitive decline compared to those without noticeable cognitive decline, we further compared score changes between those with and without noticeable cognitive decline at the second assessment while controlling for baseline score using an ANCOVA. Again, total scores and scores for the memory, executive function, and language domains were compared. For all analyses values for η^2^ determined the overall effect size of the time point in scores.

To assess the relationships between change in CS-DS total score and change in scores from the short ABS, DLD, and KBIT-2 we performed correlational analysis using Pearson's correlation. To explore whether change in scores for the memory, executive function, and language domains predicted cognitive decline as assessed by change in KBIT-2 scores we performed regression analyses, with separate regression models for each domain.

## Results

**Figure 1:**
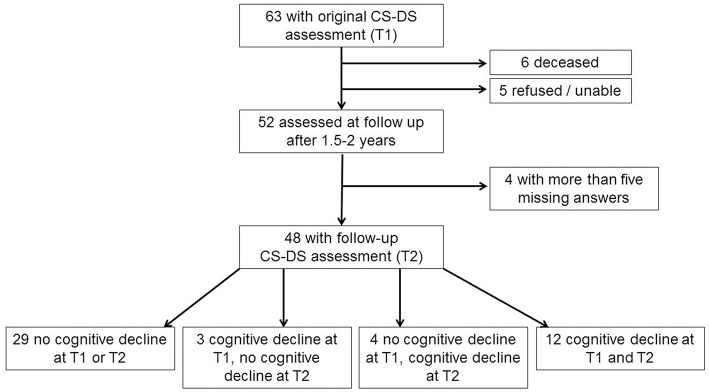
Flow chart showing participants included in longitudinal analysis.

Of the original 63 participants aged 36 years and over with a complete CS-DS at the original assessment, when contacted for follow-up six were deceased and five refused or were unable to take part. Two participants had developed advanced dementia, being fully dependent on others, and so the questionnaire was not completed as many questions were not applicable and would have missing answers. Of the 50 follow-up questionnaires completed, two contained more than five missing answers, and so were excluded from analyses. A total of 48 participants were therefore included in the longitudinal analyses. Forty seven adults had genetically confirmed triplication of chromosome 21, with trisomy 21 in 44 individuals, a translocation in one individual, and mosaic DS in two individuals. Considering the presence of noticeable cognitive decline based on information from the CAMDEX-DS, 29 participants (60.4%) showed no cognitive decline at either time point, while 12 participants (25.0%) showed cognitive decline at both time points. Four participants (8.3%) showed cognitive decline at the second time point but not the first, while three participants (6.3%) showed cognitive decline at the first time point but not the second [see Figure 1 for participant flow diagram and Table 1 for demographic information and assessment scores for participants; the full dataset for the study is available at Startin et al. (23)].

**Table 1:** Demographic information and assessment scores for participants included in longitudinal analysis.

	**Participants**
Number	48
Age at first assessment	46.06 ± 6.94 (36, 64)
Sex	26 (54.2%) male, 22 (45.8%) female
Pre-dementia level of ID	23 (47.9%) mild, 17 (35.4%) moderate, 8 (16.7%) severe
Interval between CS-DS assessments (months)	21.40 ± 2.05 (17, 24)
CS-DS total score time 1	78.83 ± 23.85 (23, 117)
CS-DS total score time 2	73.83 ± 25.35 (21, 118)
CS-DS total score change	5.00 ± 16.61 (−29, 38)
CS-DS memory score time 1	22.19 ± 8.03 (1, 32)
CS-DS memory score time 2	20.81 ± 8.63 (4, 32)
CS-DS memory score change	1.38 ± 5.01 (−7, 13)
CS-DS executive function score time 1	46.40 ± 13.59 (15, 68)
CS-DS executive function score time 2	43.54 ± 13.60 (16, 69)
CS-DS executive function score change	2.85 ± 9.76 (−18, 21)
CS-DS language score time 1	10.25 ± 4.36 (3, 18)
CS-DS language score time 2	9.48 ± 4.94 (1, 18)
CS-DS language score change	0.77 ± 4.02 (−9, 8)
Short ABS score change (*n* = 39)	6.23 ± 15.06 (−19, 55)
DLD cognitive score change (*n* = 43)	−2.84 ± 7.49 (−24, 9)
DLD social score change (*n* = 45)	−1.11 ± 7.82 (−33, 13)
KBIT-2 score change (*n* = 37)	2.65 ± 11.20 (−20, 38)

*Values show mean ± standard deviation (minimum, maximum). CS-DS total scores and scores for the executive function domain were significantly lower at time 2 compared to time 1 [t_(47)_ = 2.09, p = 0.042 and t_(47)_ = 2.03, p = 0.048, respectively]. Scores for the memory domain trended to being significantly lower at time 2 [t_(47)_ = 1.90, p = 0.064] and there was no significant change in scores for the language domain [t_(47)_ = 1.33, p = 0.191]. All comparisons were performed using paired samples t-tests. CS-DS, Cognitive Scale for Down Syndrome; DLD, Dementia Questionnaire for People with Learning Disabilities; ID, intellectual disability; KBIT-2, Kaufman Brief Intelligence Test 2; short ABS, short Adaptive Behavior Scale*.

Comparing CS-DS scores at the two time points across all participants, total scores were significantly lower at time 2 [*t*_(47)_ = 2.09, *p* = 0.042, η^2^ = 0.085, 95% CI (0.18, 9.82)]. Scores for the executive function domain were significantly lower at time 2 [*t*_(47)_ = 2.03, *p* = 0.048, η^2^ = 0.080, 95% CI (0.02, 5.69)], while scores for the memory domain trended to being significantly lower at time 2 [*t*_(47)_ = 1.90, *p* = 0.064, η^2^ = 0.071, 95% CI (−0.08, 2.83)] and there was no significant change in scores for the language domain [*t*_(47)_ = 1.33, *p* = 0.191, η^2^ = 0.036, 95% CI (−0.40, 1.94)].

Comparing CS-DS score changes between those with and without noticeable cognitive decline at the follow-up assessment, there was a trend to a significantly greater change in total score for adults with noticeable cognitive decline [*F*_(1,45)_ = 3.53, *p* = 0.067, η^2^ = 0.073]. Change in executive function score was significantly greater for those with noticeable cognitive decline [*F*_(1,45)_ = 5.17, *p* = 0.028, η^2^ = 0.103], with no significant difference in change in memory [*F*_(1,45)_ = 1.02, *p* = 0.318, η^2^ = 0.022] or language [*F*_(1,45)_ = 2.32, *p* = 0.135, η^2^ = 0.049] scores between the groups (Table 2).

**Table 2:** Change in CS-DS scores for those with and without noticeable cognitive decline at the second time point.

	**Noticeable cognitive decline**	**No noticeable cognitive decline**
CS-DS total score change	7.81 ± 16.41	3.59 ± 16.79
CS-DS memory score change	1.50 ± 4.84	1.31 ± 5.17
CS-DS executive function score change	5.13 ± 9.22	1.72 ± 9.97
CS-DS language score change	1.19 ± 3.62	0.56 ± 4.25

*Values show mean ± standard deviation. CS-DS total score changes trended to being significantly greater for adults with noticeable cognitive decline compared to those without noticeable cognitive decline [F_(1, 45)_ = 3.53, p = 0.067], with score changes for the executive function domain being significantly greater for those with noticeable cognitive decline [F_(1, 45)_ = 5.17, p = 0.028], and no significant difference in score changes for the memory or language domains [F_(1, 45)_ = 1.02, p = 0.318 and F_(1, 45)_ = 2.32, p = 0.135, respectively] between the groups. All comparisons were performed using ANCOVAs with baseline score included as a covariate. CS-DS, Cognitive Scale for Down Syndrome*.

Investigating the relationships between change in CS-DS scores and change in scores from the short ABS, DLD, and KBIT-2, change in CS-DS total score was significantly correlated with change in scores for each of the other measures (Table 3). Change in score for each of the memory, executive function, and language domains independently predicted change in KBIT-2 scores, with CS-DS memory score change predicting greater variance in KBIT-2 score change (memory: *R*^2^ = 0.28, unstandardized B = 1.23, 95% CI (0.55, 1.92), standardized beta = 0.53, *p* = 0.001; executive function: *R*^2^ = 0.15, unstandardized B = 0.48, 95% CI (0.09, 0.87), standardized beta = 0.39, *p* = 0.016; language: *R*^2^ = 0.15, unstandardized B = 1.13, 95% CI (0.20, 2.05), standardized beta = 0.39, *p* = 0.018).

**Table 3:** Correlations for CS-DS total score change with short ABS, DLD, and KBIT-2 score changes.

	**CS-DS total score change**
Short ABS score change	0.44, 0.005
DLD cognitive score change	−0.48, 0.001
DLD social score change	−0.50, <0.001
KBIT-2 score change	0.48, 0.003

*Values show Pearson's correlation coefficient, p-value. For the DLD higher scores are associated with poorer abilities, for all other measures higher scores are associated with higher abilities. CS-DS, Cognitive Scale for Down Syndrome; DLD, Dementia Questionnaire for People with Learning Disabilities; KBIT-2, Kaufman Brief Intelligence Test 2; short ABS, short Adaptive Behavior Scale*.

## Discussion

We assessed the longitudinal change in CS-DS scores in adults with DS aged 36 years and over across two time points, ~1.5–2 years apart. Total scores showed a significant decrease over this time, with scores in the executive function domain also showing a significant decrease and scores in the memory domain trending to a significant decrease. Change in total scores trended to be significantly greater in those with noticeable cognitive decline compared to those without noticeable cognitive decline at the second time point, with scores in the executive function domain only showing a significantly greater change in those with noticeable cognitive decline. Change in CS-DS total scores showed significant correlations with change in scores for informant measures of adaptive abilities and symptoms associated with cognitive decline, in addition to a participant measure of cognitive abilities. Finally, we found that change in score for the CS-DS memory domain better predicted change in participant cognitive abilities compared to the executive function or language domains.

Our results provide further support for the validity of the CS-DS as a tool to assess everyday cognitive abilities and associated decline in people with DS. We previously showed that CS-DS scores were lower for individuals with noticeable cognitive decline compared to those without noticeable cognitive decline (13). We have now extended this finding to show that the CS-DS is sensitive to detect longitudinal decline in scores, with decline in CS-DS scores associated with decline in abilities assessed using informant questionnaires and participant assessment. These changes are likely reflective of the changes adults with DS undergo as they age; the development of dementia eventually occurs in almost all adults (3), and it has been suggested that a third of adults aged over 30 may show longitudinal cognitive decline over 16 months (24). Supporting the longitudinal changes in CS-DS score detecting underlying changes associated with cognitive decline, changes in CS-DS scores showed significant correlations with changes in scores on the DLD, an informant questionnaire assessing symptoms related to dementia. Although the difference in score change for those with and without noticeable cognitive decline at the second time point did not reach significance, this was likely due to the small sample size of individuals with decline. Future studies should further explore this.

Our results also suggest the presence of noticeable cognitive decline at the second time point as assessed by informant report was associated with significantly greater decline in scores for the executive function domain only. This may suggest informants better detect changes in this domain compared to changes in the memory or language domains. In contrast, longitudinal change in scores for the memory domain better predicted change in directly assessed cognitive abilities compared to changes in scores for the executive function or language domains. This may suggest changes in assessed cognitive ability are most strongly associated with change on informant rated memory scores. It is also possible these results may indicate different domains of the CS-DS are able to detect changes at different stages of cognitive decline, with changes in the memory domain being apparent across both early and later stages of cognitive decline, and greater changes in the executive function domain only being apparent when noticeable cognitive decline has occurred. Similarly, our previous studies investigating cognitive changes across the lifespan in people with DS found changes in memory abilities assessed via either direct participant assessment or informant report were more strongly associated with the early stages of progression of dementia than changes in executive functioning (25). Other studies have suggested the earliest changes associated with cognitive decline in DS are in memory (26, 27), though some studies noted prominent changes in executive functioning (24, 28). Understanding the time course of cognitive changes with the development of cognitive decline in DS is essential to aid in the diagnosis and monitoring of dementia.

The use of additional time points in future studies, along with larger samples, will allow further exploration of longitudinal changes in CS-DS scores. This will allow a potential difference in the change in score for those with and without noticeable cognitive decline to be better determined, in addition to better exploring whether CS-DS scores may be predictive of future decline in individuals. Given the high prevalence of dementia in people with DS, it is essential to detect the earliest signs of cognitive decline to assist in the diagnosis of dementia and to improve individuals' prognosis through suitable health care. Detecting these earliest changes requires longitudinal assessments using measures sensitive to these changes. Larger studies are needed to determine whether the CS-DS may be suitable for this, and it will be essential to compare changes in CS-DS score to changes in other relevant informant questionnaires to determine which measures are most sensitive to detecting changes at the earliest stage of cognitive decline; such questionnaires include the CAMDEX-DS (21), the DLD (11), the DSQIID (Dementia Screening Questionnaire for Individuals with Intellectual Disabilities) (12), and the DSDS (Dementia Scale for Down Syndrome) (10). It is further possible that different questionnaires may be useful in measuring decline at different stages in the progression of dementia. Further, the identification of potential CS-DS score cut-offs for single and longitudinal assessments to detect cognitive decline in people with DS may be of use in clinical settings. Given the wide variability in pre-morbid level of ID in people with DS, longitudinal cut-offs or those based on level of ID would be necessary for high sensitivity, and larger studies will enable this.

The CS-DS was designed as an informant outcome measure to assess cognitive abilities, and to detect changes in cognitive abilities over time. Results presented here and in Startin et al. (13) indicate the CS-DS is a valid informant measure for cognitive abilities, and the CS-DS is sensitive to longitudinal cognitive decline in adults aged 36 years and over. However, it should be noted there is some overlap in the scores for those with and without noticeable cognitive decline, which may be potentially related to pre-morbid level of ID, and so the CS-DS alone is not suitable for diagnosing dementia. Instead, the CS-DS may be useful as a screening questionnaire for possible cognitive decline at an individual level in people with DS. It is also possible the CS-DS is sensitive to detecting improvements in cognitive abilities following interventions, and future studies are needed to investigate this.

It should however be noted that a single informant questionnaire is not able to assess all aspects of cognitive abilities, instead such questionnaires are only able to provide a broad measure of abilities. Some abilities, for example those relating to specific modalities such as visuospatial and verbal memory abilities, would be best measured by formal participant assessment, providing participants are able to engage in such an assessment, and it may not be appropriate to assess such abilities via informant report. To fully explore all aspects of cognitive abilities and associated changes in these in people with DS it would therefore be essential to undertake a detailed cognitive assessment with participants using a comprehensive cognitive battery similar to previously published batteries (5–9), with informant questionnaires such as the CS-DS complementing this and providing useful information for individuals unable to engage in assessment.

The main limitation of the current study is the relatively modest sample size, with a small number of individuals with noticeable cognitive decline. In particular, too few individuals in the present study developed noticeable cognitive decline between the two time points for subgroup analysis. Larger samples with multiple time points will allow for analysis to determine whether scores on the CS-DS can predict future decline. Another limitation was the use of another informant questionnaire (the CAMDEX-DS) to define noticeable cognitive decline for analyses, although the use of the KBIT-2 to assess the relationship between changes in CS-DS and KBIT-2 scores allowed us to assess change in cognitive abilities in participants directly. Other limitations apply to the CS-DS itself; the questionnaire may be unsuitable for individuals with severe dementia, as shown by incomplete questionnaires for such individuals. However, the CS-DS was not designed to be sensitive to later changes with dementia, and questionnaires based on the progression of dementia symptoms already exist.

Our results suggest changes in scores on the CS-DS are a valid measure to detect longitudinal changes in everyday cognitive abilities consistent with cognitive decline in adults with DS. Although larger, longer studies are needed to further evaluate this and to determine the use of the CS-DS in predicting cognitive decline, we suggest this questionnaire may be a useful addition to test batteries to measure cognitive abilities and changes in these in individuals with DS.

## Data Availability

The dataset analysed for this study can be found in the figshare repository at https://doi.org/10.6084/m9.figshare.7011632.v1.

## Ethics Statement

Ethical approval was obtained for the LonDownS study from the North West Wales Research Ethics Committee (13/WA/0194). Where individuals had capacity to consent for themselves we obtained written informed consent in accordance with the Declaration of Helsinki, including consent to collect information from informants. Where individuals did not have capacity to consent for themselves, a consultee was appointed and asked to sign a form to indicate their decision regarding the individuals' inclusion based on their knowledge of the individual and his/her wishes, in accordance with the UK Mental Capacity Act 2005.

## Author Contributions

AS conceived the adult cohort study in conjunction with LonDownS principal investigators. CS and AS designed the study and data analysis. CS, BL, SH, and RH performed data collection. CS and BL analyzed the data and drafted the initial version of the report. All authors contributed to revision and editing of the report.

### Conflict of Interest Statement

The authors declare that the research was conducted in the absence of any commercial or financial relationships that could be construed as a potential conflict of interest.
